# Reactive Carbonyl Species and Protein Adducts: Identification Strategies, Biological Mechanisms and Molecular Approaches for Their Detoxification

**DOI:** 10.3390/antiox10050690

**Published:** 2021-04-28

**Authors:** Giancarlo Aldini, Alessandra A. Altomare

**Affiliations:** Department of Pharmaceutical Sciences, Università degli Studi di Milano, Via L. Mangiagalli 25, 20133 Milan, Italy; alessandra.altomare@unimi.it

The Special issue is composed of 13 contributions: 9 research papers and 4 reviews. With regard to the reviews, two of them are highly comprehensive on the chemistry and biological effects of RCS and protein carbonylation and the other two are more targeted on specific subjects involving protein carbonylation. In particular, the review by Shivkanya Fuloria, et al. [1] is a very clear and detailed review of the chemistry (source, reaction, methods of measurement) and biological effects of RCS and their reaction products with proteins. Those who are not very familiar with the topic of the Special Issue should read this review first.

The review by Álvaro Viedma-Poyatos, et al. [2] is another very clear and comprehensive review of RCS and protein carbonylation and gives the basic aspects of protein lipoxidation in terms of reaction mechanisms and functional effects. This review well integrates the previous one since protein lipoxidation is treated not only as a harmful process but also as a potential regulatory process which appears to be involved in cellular defence responses and adaptation to stress. This aspect is of great interest, especially considering that protein lipoxidation, although not enzymatically regulated, seems not to be a random process but more likely is characterized by selectivity. We strongly believe that this aspect will be largely debated and will represent a research arena in the near future. 

The two other reviews focus on more specific subjects. In particular, the paper by Alba Rodríguez-García, et al. [3], describes the relationship of RCS and protein carbonylation in hematological malignancies, with a particular insight into their role in disease development, and potential implications for treatment. 

The last review by Mariona Jové, et al. [4] regards the chemistry and biology of the adduct between malondialdehyde (MDA), one of the most abundant lipid-peroxidation by-products, and lysine (MDA-Lys). The specificity of MDA-Lys formation in proteins, the methodology used for its detection and quantification, the metabolism of MDA-modified proteins, and the detrimental effects of this protein modification are clearly detailed and reviewed. Based on the findings, the authors propose and sustain MDA-Lys as an indicator of aging.

One of the limiting aspects in the research of RCS–protein adducts is the difficulty of their identification, characterization and quantification; this is due to the fact that these adducts are very heterogeneous and usually present in negligible amounts. Beside ELISA and Western Blot methods that furnish a quite valuable although general index of protein carbonylation, more sensitive and specific analytical techniques such as mass spectrometry coupled to a nano-LC system are currently used for profiling RCS–protein adducts. The research in this field is very attractive because we are still searching for suitably robust, selective and sensitive methods. In line with this interest, four of the nine research papers of the Special Issue regard analytical and mass spectrometry-based methods for profiling protein carbonylation. 

In particular, in the paper by Carolina Luna, et al. [5], the authors monitor the formation of allysine and α-aminoadipic acid (α-AA) in a glyco-oxidative condition, using albumin as protein substrate. While both allysine and α-AA were found to increase following the increase of glucose concentrations, the carboxylic acid was only detected at pathological glucose concentrations and appeared to be a more reliable indicator of glyco-oxidative stress. 

Analysis of protein carbonyls, and in particular identification and characterization of the adducted moiety and site of modifications, represents a very difficult challenge due to the fact that the modifications are usually present in a very reduced concentration in respect to the native proteins, and are quite heterogeneous and unstable. The paper by Alessandra Altomare, et al. [6] reports a mass spectrometric approach for the identification of a set of albumin adducts which were found to be significantly higher in heart failure (HF) patients with respect to healthy subjects. The paper, besides reporting an analytical approach to identify protein adducts by an untargeted approach, also reports glyoxal as a carbonyl species whose formation is increased in HF patients, and which results from an overproduction of glyoxal and/or reduction in its metabolic removal.

A methodological paper on protein carbonylation analysis by LC–MS is then reported by Juan Camilo Rojas Echeverri, et al [7]. The paper reports a workflow which addresses several analytical issues on sample preparation and data analysis, and which can significantly improve the identification of many reactive carbonyl PTMs and reproducibly quantitate the corresponding peptides. The group of Prof. Hoffman [8] has contributed with a second paper by Michele Wölk. By using a bottom-up proteomics approach and a combination of data-independent and data-dependent acquisition methods, a fully detailed profiling of low-molecular-weight carbonyls and protein modifications in flavored milk was reported. The method found 26 different PTMs related to glycation, AGE-formation, carbonylation, and lipid oxidation and additionally 13 sample-specific LMW carbonyl adducts in the bovine milk proteome.

The remaining five research papers focus on a very stimulating and debated topic, the functional effects of RCS and protein carbonylation in physio-pathological conditions. It is now well established that an excessive formation of RCS, leading to protein adduction, contributes to the pathogenetic mechanisms of some oxidative based diseases. Moreover, the protective effect of such compounds when formed in a low concentration is now also recognized, underlining their hormetic activity. The following papers have deeply investigated such important aspects.

The paper by Audrey Swiader, et al. [9] gives strong evidence of the involvement of 4-hydroxynonenal (HNE), a well-known RCS lipid peroxidation by-product, formed in the skin by UV irradiation, in promoting fibroblast senescence in skin photoaging. Furthermore, vimentin was found the be the target of HNE adduction and carnosine, a well-known sequestering agent of HNE, a compound able to prevent both vimentin modification and fibroblast senescence, thus on the one hand confirming the involvement of HNE in skin aging and on the other the potential beneficial effect of the dipeptide.

The function study of HNE is also addressed in the paper by Julia Keller, et al. [10]. The paper demonstrates that HNE formed in the intestinal tract by the reaction of dietary heme iron (meat) and lipids, besides acting locally, is also absorbed forming protein adducts in heart, liver and skeletal muscle tissues. On the basis of the results, the authors conclude that red meat over-consumption, and more largely peroxidation-prone food consumption, could have major consequences on the onset/development of chronic diseases through the formation and absorption of lipid-peroxidation derived carbonyl species.

In the paper by Amy K. Hauck, et al. [11], the effects of HNE and 4-hydroxyhexenal (HHE) in modifying histones in in vitro and in vivo conditions have been reported by proteomic studies. Histones were found as specific targets of carbonylation and are differentially modified by the two reactive lipid species HNE and HHE. Furthermore, the authors reported that the modifications accumulate in adipose tissue as a consequence of high-fat feeding (HFD) and of aging.

Besides HNE, methylglyoxal (MGO), a side product of glycolysis, is another RCS whose biological effects have been investigated in the present Special Issue by two research papers.

The paper by Martina Hüttl, et al. [12] investigates the effect of MG on metabolic changes in the visceral adipose tissue of hereditary hyper-triglyceridaemic rats, a non-obese model of metabolic syndrome. The functional effect is explored by a transcriptome and gene expression profiling. The results clearly indicate the effect of MG in adipose tissue dysfunction by impairing adipose tissue insulin sensitivity and stimulating the inflammatory response at both transcriptome and metabolic levels.

A quite interesting role of MGO in glucose and lipid metabolism in spontaneously hypertensive rats (SHR) was then reported by Jan Šilhavý, et al. [13]. MGO, as well as other dicarbonyls, are detoxified by the glyoxalase system in which glyoxalase 1 (Glo1) serves as the rate-limiting enzyme. In this study, MG tissue increase, induced by Glo1 gene downregulation, resulted in positive effects; in particular, decreased serum triglycerides and a reduced adiposity and ectopic fat accumulation in the heart, which was associated with AMPK activation. The paper by Jan Šilhavý and that by Julia Keller underline that RCS, in this case of MGO, are not just toxic metabolites but that at certain physiological concentrations may play a role in regulating glucose and lipid metabolism. 

In conclusion, the Special Issue introduced here reports a collection of review and research papers on the different aspects of RCS and protein carbonylation, among which their hormetic activity, which we believe is a very stimulating and expanding subject field of oxidative stress. 
